# A novel tRNA-derived fragment, tRF-19-79MP9PJZ, promotes uterine corpus endometrial carcinoma progression by targeting DSC3

**DOI:** 10.1007/s13577-026-01432-x

**Published:** 2026-08-06

**Authors:** Huabo Jiang, Ting Jiang, Yuting Shen, Hongyan Jiang, Qing Yuan

**Affiliations:** 1 Department of Gynecology, Shanghai Key Laboratory of Maternal Fetal Medicine, Shanghai Institute of Maternal-Fetal Medicine and Gynecologic Oncology, Shanghai First Maternity and Infant Hospital, School of Medicine, Tongji University, No. 2699 Gaoke West Road, Shanghai, 200092 China; 2 Department of Gynecology, Obstetrics and Gynecology Hospital, Fudan University, Shanghai, 200011 China; 3 Department of Gynecology, Obstetrics and Gynecology Hospital, Fudan University, Yangtze River Delta Integration Demonstration Zone (Qingpu), Shanghai, 201713 China; 4 Department of Gynecology and Obstetrics, Maternal and Child Health Hospital, Linwu, 424300 China

**Keywords:** tRFs, tRF-19-79MP9PJZ, DSC3, Uterine corpus endometrial carcinoma, Molecular mechanisms

## Abstract

Uterine corpus endometrial carcinoma (UCEC) ranks as the 6th most common malignancy among women. Emerging evidence indicates that the dysregulation of tRNA-derived fragments (tRFs) is involved in the pathogenesis of multiple cancer types, including UCEC; however, the molecular mechanisms underlying the roles of tRFs in UCEC remain poorly characterized. Desmocollin3 (DSC3), a transmembrane protein, is predominantly expressed in the basal and suprabasal layers of normal stratified epithelia. While accumulating evidence has implicated DSC3 in the pathogenesis of multiple disease entities, its functional role in UCEC remains elusive. The present study is designed to investigate the functional significance and underlying molecular mechanisms of tRF-19-79MP9PJZ in the progression of UCEC. In this study, tRF-19-79MP9PJZ was found to be significantly upregulated in UCEC tissues and cell lines, with its elevated expression correlating with unfavorable prognostic outcomes in UCEC patients. Furthermore, tRF-19-79MP9PJZ knockdown was observed to suppress the proliferative and migratory capacities of UCEC cells, while concurrently enhancing apoptotic processes. At the mechanistic level, tRF-19-79MP9PJZ was demonstrated to facilitate UCEC progression through targeted regulation of DSC3. Collectively, this study elucidates a previously uncharacterized mechanism whereby tRF-19-79MP9PJZ drives UCEC development, thereby highlighting the potential of the tRF-19-79MP9PJZ/DSC3 axis as a therapeutic target for UCEC intervention.

**Supplementary Information:**

The online version contains supplementary material available at 10.1007/s13577-026-01432-x.

## Introduction

Uterine corpus endometrial carcinoma (UCEC) represents the 6th most common malignancy in females, with 417,000 new cases documented globally in 2020 [1]. The lifetime risk of developing UCEC is approximately 3% among women, with a median age at diagnosis of 61 years [1, 2]. Over the past three decades, the incidence of UCEC has increased by 132%, with the highest incidence rates reported in North America, followed by Eastern and Central Europe [1]. Notably, the number of cases among women under 40 years of age has doubled, accounting for 4.2% of all low-grade UCEC diagnoses in the USA [1, 3]. Accordingly, despite the global rise in age-standardized incidence rates, the most substantial increase in UCEC cases has been observed in high-income countries [4]. While over 95% of early-stage UCEC cases are curable, a considerable proportion of UCEC is diagnosed at advanced stages, which is associated with a 5-year survival rate of less than 20% [5, 6]. This scenario underscores the critical importance of identifying effective early diagnostic biomarkers and gaining a comprehensive understanding of UCEC pathogenesis to facilitate the advancement of targeted therapeutic strategies.

Non-coding RNAs in cancer have attracted substantial research interest in recent years [7–10]. tRNA-derived fragments (tRFs) represent a class of small single-stranded non-coding RNAs generated via endonucleolytic cleavage of precursor or mature transfer RNA (tRNA) molecules [11]. Characterized by a length of 12 to 40 nucleotides, tRFs were once considered mere byproducts of tRNA degradation [11, 12]. These molecules are categorized into four subgroups: tRF-1, tRF-3, tRF-5, and i-tRFs [12]. tRFs exert important roles in multiple molecular processes, including RNA and protein biosynthesis as well as oncogenic transformation [12]. Aberrant expression of tRFs is associated with the proliferation, migration, and invasiveness of specific cancer cells, including those of UCEC [13–16]. Furthermore, existing literature has documented that tRF-20-S998LO9D exerts inhibitory effects on EC cells via the upregulation of SESN2 [17]. AS-tDR-007333 facilitates the malignant progression of non-small cell lung cancer (NSCLC) through the regulation of HSPB1/MED29 and ELK4/MED29 signaling axes [16], and tRF-19-W4PU732S promotes the malignant phenotype of breast cancer cells by inhibiting RPL27A [18]. Nevertheless, despite accumulating evidence suggesting that tRFs, similar to well-characterized microRNAs, may exert their functions through targeted regulation of downstream genes [12, 16, 17], the precise molecular mechanisms underlying the roles of tRFs in UCEC remain to be elucidated.

In the present study, we identified a novel tRF, designated as tRF-19-79MP9PJZ, and validated its upregulation in UCEC as well as its association with the survival outcomes of UCEC patients. Furthermore, we demonstrated that tRF-19-79MP9PJZ promotes UCEC progression through targeted regulation of desmocollin-3 (DSC3). These findings contribute to a deeper understanding of the pathogenic mechanisms underlying UCEC and may provide potential novel biomarkers and therapeutic targets for its diagnosis and treatment.

## Materials and methods

### Reagents

Dulbecco’s Modified Eagle Medium (DMEM) and fetal bovine serum (FBS) were purchased from Gibco (USA). The First Strand cDNA Synthesis Kit and Lipofectamine 3000 were obtained from Thermo Fisher Scientific, Inc. (USA). Matrigel was sourced from BD (USA). The Dual Luciferase Reporter Assay Kit was acquired from Yeasen (China). Radio-immunoprecipitation assay (RIPA) buffer was purchased from Beyotime (China). The Cell Counting Kit-8 (CCK8) assay kit was obtained from Dojindo Corporation (Japan). The antibody against GAPDH was purchased from Abcam (USA). The NEBNext® Multiplex Small RNA Library Prep Set for Illumina® kit was sourced from New England BioLabs (USA). The antibody against DSC3 was acquired from Acris (Germany). TRIzol® reagent was purchased from Invitrogen (USA).

### Dataset from TCGA

tsRNA profiles specific to UCEC were retrieved from the OncotRF Database (http://bioinformatics.zju.edu.cn/OncotRF/) [19]. Transcriptomic data from UCEC tissues and normal endometrial tissues were processed and analyzed using DESeq2 (R package version 1.32) within R software (version 4.0.1). tsRNAs with a median read-count of less than 10 were excluded from subsequent analyses. Differential expression analysis was performed to identify tsRNAs meeting the criteria of adjusted *p* < 0.05 and |logFC|> 1, which were selected for downstream analysis.

### Clinical samples

The study protocol was approved by the Medical Ethics Committee of Shanghai First Maternity and Infant Hospital (approved number: [IRB: H-1126–074]). Written informed consent was obtained from each participant prior to enrollment. Tumor tissues and adjacent non-tumor tissues were collected from 40 patients with pathologically confirmed UCEC who were registered at Shanghai First Maternity and Infant Hospital. The harvested tissues were immediately preserved in liquid nitrogen for short-term storage or transferred to a – 80 °C freezer for long-term preservation.

### Cell culture and transfection

Human normal endometrial stromal cells (ESCs) and human UCEC cell lines (HEC1A, HEC-1-B, and Ishikawa) were obtained from the American Type Culture Collection (ATCC, USA). These cells were maintained in DMEM supplemented with 10% FBS, 100 U/mL penicillin, and 100 μg/mL streptomycin, and cultured in a humidified incubator at 37 °C in 5% CO_2_.

Small interfering RNA targeting tRF-19-79MP9PJZ (si-tRF), DSC3 (si-DSC3), and their negative controls (si-NC and control), tRF-19-79MP9PJZ mimics, the corresponding negative control (tRF-NC), were purchased from GenePharma (China). HEC1A and Ishikawa cells were transfected with each construct at a final concentration of 50 nM using Lipofectamine 3000 reagent (Thermo Scientific, USA) following the manufacturer’s protocol. Transfected cells were harvested for subsequent experiments after a 48-h incubation period.

### tRFs sequencing

Total RNA was extracted using TRIzol® reagent. To minimize RNA modifications that might interfere with small RNA library construction, initial pretreatments were conducted, including the following steps: 3’-aminoacyl deacylation to generate 3’-OH groups (to enable 3’-adaptor ligation), removal of 3’-cP (2’,3’-cyclic phosphate) to form 3’-OH groups, phosphorylation of 5’-OH to 5’-P (to facilitate 5’-adaptor ligations), and demethylation of m^1^A and m^3^C residues to promote efficient reverse transcription. RNA samples were subjected to library preparation using the NEBNext® Multiplex Small RNA Library Prep Set for Illumina® kit. This process included 3’ and 5’ adapter ligation, cDNA synthesis, and subsequent PCR amplification. High-throughput sequencing was conducted on an Illumina NextSeq 500 system (Illumina, USA) at Aksomics Inc. (China) using the NextSeq 500/550 V2 kit (#FC-404–2005, Illumina).

Sequencing quality was evaluated using FastQC software. Trimmed reads (passing Illumina quality filters and having 3’-adaptor sequences removed using Cutadapt) were aligned to mature and precursor tRNA sequences from the tRNA database (http://GtRNAdb.ucsc.edu) using NovoAlign software. Unmapped reads were further aligned to other relevant nucleic acid databases, including those for mRNA, rRNA, snRNA, snoRNA, piRNA, and miRNA. The expression levels of tRFs were quantified and normalized to transcripts per million (TPM) of total aligned tRNA reads. A *p* value < 0.05 was considered statistically significant.

### Target gene prediction for tRF-19-79MP9PJZ

tRFs represent a newly recognized class of small non-coding RNAs with numerous uncharacterized biological functions. Currently, it is well established that tRFs may exert regulatory roles and harbor target molecules analogous to those of miRNAs [16, 20–22]. Accordingly, we employed tsRFun (https://rna.sysu.edu.cn/tsRFun/) [23] to predict the downstream target genes of tRF-19-79MP9PJZ. The correlation between tRF-19-79MP9PJZ and its putative target genes was assessed using Spearman’s rank correlation test.

### Quantitative real-time PCR (qRT-PCR) assay

Total RNA was reverse transcribed using the First Strand cDNA Synthesis Kit. qRT-PCR was performed using the Universal SYBR Green Master Mix on a 7500 Fast Real-Time PCR system (Applied Biosystems, USA) to assess the expression levels of target RNAs. GAPDH was used as an internal control for normalization, and relative expression levels were calculated using the 2^−ΔΔCt^ method. The primer sequences used for amplification are as follows:

DSC3 forward, 5’-GAAAGTAGTAGACCTGGTACT-3’;

DSC3 reverse, 5’-ACGCCTGTGCTGGGATGCA-3’;

GAPDH forward, 5’-TGTTGTGGATCTGACCTGCC-3’;

GAPDH reverse, 5’-AAGTCGCAGGAGACAACCTG-3’.

The stem-loop RT primers and quantitative PCR primers for tRF-19-79MP9PJZ were designed and synthesized by Aksomics (Shanghai, China) based on its specific mature sequence GTTTCCGTAGTGTAGTGGT.

### Cell proliferation evaluation

Cell viability was assessed using the CCK-8 assay in accordance with the manufacturer’s protocol. Briefly, CCK8 reagent was added to cells cultured in 96-well plates at a volume of 10 μL per well (corresponding to 1000 cells per well), followed by incubation in the dark at 37 ℃ for 4 h. Subsequently, the absorbance at 450 nm was measured using a microplate reader (BioTek Elx800—BioTek Instruments, Inc., Winooski, USA).

### Transwell assay

The migration capacity of cells was evaluated using a 24-well Transwell chambers with 0.8 μm pores, which were not coated with Matrigel. Cells were seeded into the upper chamber of the Transwell device, while the lower chamber was filled with a chemoattractant (10% FBS) to induce migration across the membrane. Following incubation for the specified duration, the migrated cells were fixed, subsequently stained with 0.1% crystal violet, and imaged for quantitative analysis.

### Western blotting analysis

Total proteins were extracted using RIPA buffer supplemented with protease inhibitors. Subsequently, 20 μg of protein samples were denatured in a sample buffer containing beta-mercaptoethanol, followed by separation via polyacrylamide gel electrophoresis (PAGE) and transfer onto a polyvinylidene difluoride (PVDF) membrane through electroblotting. After blocking with 5% bovine serum albumin (BSA) in Tris-buffered saline with Tween® 20 (TBST) for 1 h at room temperature, the membrane was incubated with primary antibodies and subsequently secondary antibodies. Protein signals were detected using a chemiluminescent substrate and visualized using a chemiluminescence imaging system.

### Dual-luciferase reporter gene

HEC1A and Ishikawa cells were seeded in 24-well plates and co-transfected with tRF-19-79MP9PJZ mimics (tRF-mimics) or their negative control (tRF-NC), together with psiCHECK-2 plasmids harboring either wild-type DSC3 sequences or mutated DSC3 sequences, using Lipofectamine 3000 reagent. Following a 48-h incubation period, luciferase activities (both Firefly and Renilla luciferase) were measured using the Dual-Luciferase Reporter Assay kit.

### RNA immunoprecipitation (RIP) assay–qPCR

RIP assay was performed in accordance with the protocol provided by the Magnetic RIP kit. The cells were incubated overnight at 4 °C with either anti-AGO2 antibodies or anti-IgG antibodies (as a control) to immunoprecipitate intracellular protein–RNA complexes. Subsequently, the cells were treated with Proteinase K, followed by RNA extraction. Nonspecific adsorption to magnetic beads was eliminated through stringent RIP washing steps. Finally, the immunoprecipitated RNAs were quantified using qPCR.

### Animal experiment

The animal experiments were conducted in accordance with the ARRIVE guidelines and approved by the Animal Ethical and Welfare Committee of Shanghai First Maternity and Infant Hospital (approval number: [FM-IH-20240727]). Twelve 4-week-old BALB/C nude mice were randomly divided into two groups (n = 6 per group). HEC1A cells (3 × 10^5^ cells per mouse) with or without tRF-19-79MP9PJZ knockdown were subcutaneously injected into the mice to establish subcutaneous xenografts models. In strict accordance with the principles of animal welfare, anesthesia was used to relieve pain during euthanasia. The mice were euthanized using sodium pentobarbital injected intraperitoneally at a dose of 150 mg/kg, and death was confirmed by the cessation of the heartbeat. The volume and weight of the subcutaneous tumors were monitored. Tumor volumes were measured on days 7, 14, 21, 28, and 35, while tumor weight was recorded on day 35.

### Statistics

Statistical analyses were performed using SPSS 22.0 software (Chicago, USA). A two-tailed Student’s *t* test was used for comparisons between two groups, whereas Chi-square test was applied for comparisons involving multiple groups. For survival analyses, patients were stratified into two groups based on the median expression level of the target tsRNA as the cutoff value. Differences in overall survival (OS), disease-free survival (DFS), and relapse-free survival (RFS) between the two groups were analyzed using the survival package (version 3.1) in R software, with the log-rank test employed for statistical comparison. A *p* value < 0.05 was considered statistically significant.

## Results

### tRF-19-79MP9PJZ is upregulated in UCEC and is a prognostic factor for UCEC patients

**Fig. 1:**
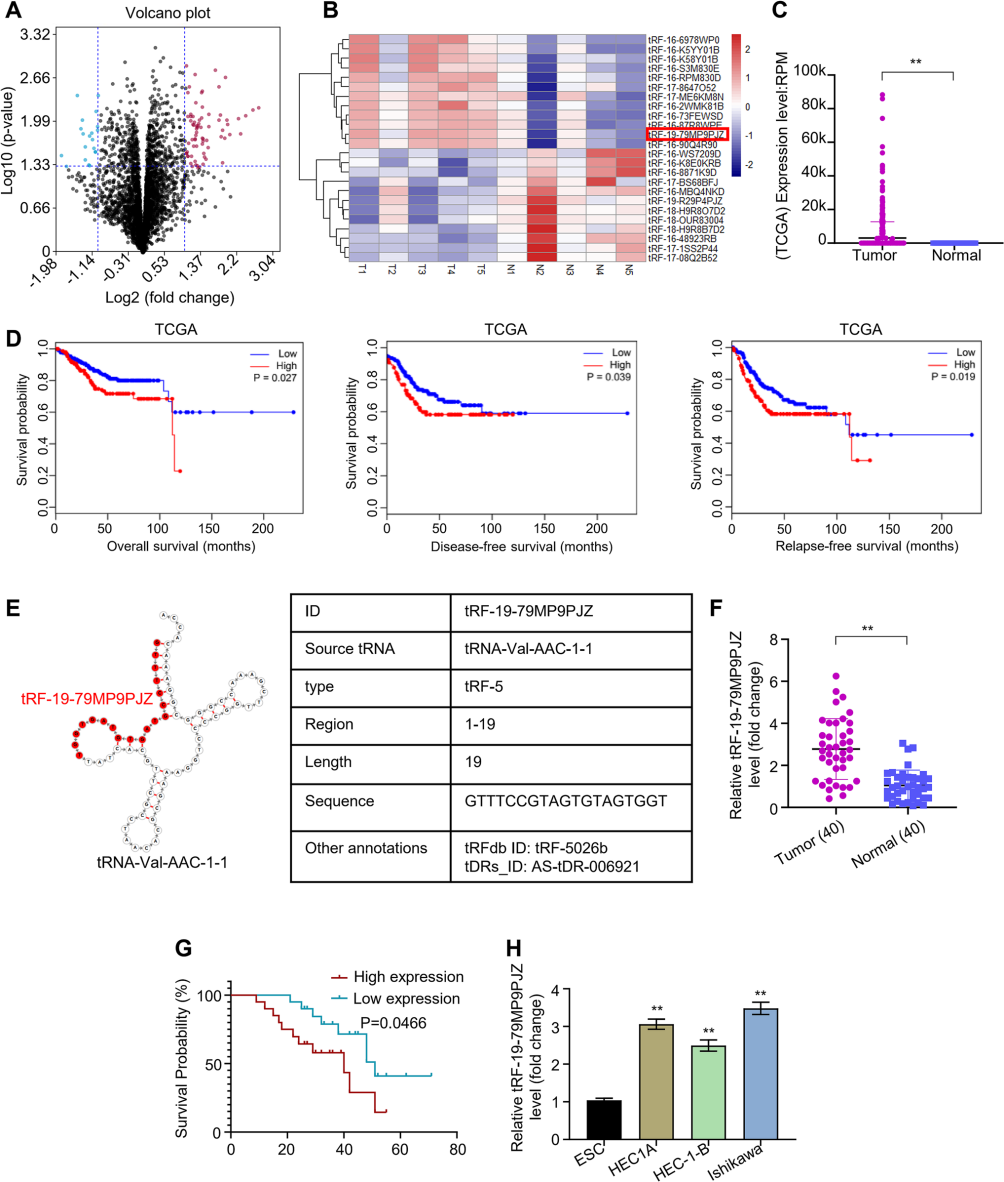
Expression analysis of tRF-19-79MP9PJZ in UCEC. **A** Volcano plot illustrating differentially expressed tRFs between adjacent non-tumor tissues and tumor tissues (*n* = 5). **B** Heatmap showing the top 24 differentially expressed tRFs (12 upregulated and 12 downregulated) between adjacent non-tumor tissues and tumor tissues (*n* = 5). |logFC|> 1, *p* < 0.05. **C** Analysis of the TCGA database revealing high expression of tRF-19-79MP9PJZ in UCEC tumor tissues (Tumor, *n* = 546; Normal, *n* = 33). **D** TCGA database analysis indicating that patients with high tRF-19-79MP9PJZ expression generally exhibit poor prognosis. **E** tRF-19-79MP9PJZ is derived from tRNA-Val-AAC-1–1 and classified as a tRF-5 subtype. **F** qRT-PCR analysis of tRF-19-79MP9PJZ expression in tumor tissues and adjacent non-tumor tissues from 40 UCEC patients (*n* = 40). **G** Kaplan–Meier curve depicting overall survival of 40 UCEC patients, with the median expression of tRF-19-79MP9PJZ used as the cutoff value (*n* = 40). **H** qRT-PCR analysis of tRF-19-79MP9PJZ expression in UCEC cell lines (HEC1A, HEC-1-B, and Ishikawa) and ESC. **p < 0.01

To investigate the differentially expressed tRFs in UCEC, we initially compared tRF expression levels between five pairs of UCEC tumor tissues and their adjacent non-tumor tissues. The comparison revealed that tRF-19-79MP9PJZ was significantly upregulated in tumor tissues. Volcano plot analysis (Fig. 1A) and heatmap visualization (Fig. 1B) illustrate the differential expression profiles, with the screening criteria of |logFC|> 1 and *p* < 0.05. Specifically, 42 upregulated tRFs and 21 downregulated tRFs were identified (Fig. 1A). The top 24 differentially expressed tRFs (12 upregulated and 12 downregulated), including tRF-19-79MP9PJZ, were highlighted in the heatmap (Fig. 1B). To select a specific candidate for downstream functional characterization, we applied a strict multi-step filtering strategy. First, considering our hypothesis to identify an oncogenic tRF that potentially inhibits tumor suppressors, we restricted our focus to the top 12 upregulated tRFs with sufficient basal expression abundance. Second, we cross-referenced these 12 candidates with the large-scale TCGA UCEC cohort to evaluate their comparative clinical significance. As detailed in Table S1, we evaluated the prognostic value of all top 12 up-regulated tRFs. Among them, tRF-19-79MP9PJZ uniquely emerged as the most promising target, exhibiting the most significant correlation with poor prognosis (p = 0.027). As shown in our subsequent validation, it was consistently highly expressed in UCEC tumor tissues (Fig. 1C), and its upregulation uniquely correlated with poorer prognosis in UCEC patients (Fig. 1D). Finally, preliminary bioinformatic analysis indicated its robust potential to target critical cancer-associated genes, including DSC3. Based on this rigorous, hypothesis-driven selection funnel, tRF-19-79MP9PJZ was selected as the primary focus of this study. Analysis using tsRFun revealed that tRF-19-79MP9PJZ (which directly corresponds to the MINTbase license plate identifier) is derived from the 5’-end of the mature tRNA-Val–AAC-1–1, belonging to the tRF-5 subtype with a length of 19 nucleotides (5’-GTTTCCGTAGTGTAGTGGT-3’) (Fig. 1E). To facilitate cross-database referencing and reproducibility, we systematically annotated this sequence across major repositories. The identifier tRF-19-79MP9PJZ corresponds to its MINTbase license plate. In tRFdb, it is designated as tRF-5026b. While an exact match was not currently cataloged in tsRBase, we applied the highly standardized tDRnamer system, yielding the identifier AS-tDR-006921. This classification contributes to our understanding of its biogenesis and potential roles in UCEC pathogenesis. Furthermore, qRT-PCR verified the high expression of tRF-19-79MP9PJZ in a cohort of 40 UCEC patients (Fig. 1F). Such robust validation with a relatively large sample size supports the potential of tRF-19-79MP9PJZ as a diagnostic or prognostic indicator for UCEC. Using the median expression level of tRF-19-79MP9PJZ as a cut-off value, the 40 patients were stratified into high-expression and low-expression groups. The clinical outcomes, including the OS, DFS, and RFS, indicated that patients with high tRF-19-79MP9PJZ expression exhibited a poor prognosis (Fig. 1G). Additionally, qRT-PCR analysis showed that the expression level of tRF-19-79MP9PJZ was significantly elevated in UCEC cell lines (HEC1A, HEC-1-B, and Ishikawa) compared with normal human endometrial epithelial cells (ESC) (Fig. 1H). Collectively, these findings suggest that tRF-19-79MP9PJZ is a promising candidate for further investigation into its role in UCEC pathogenesis and its clinical significance.

### tRF-19-79MP9PJZ-knockdown inhibits proliferation and migration ability and promotes apoptosis in UCEC cell lines

**Fig. 2:**
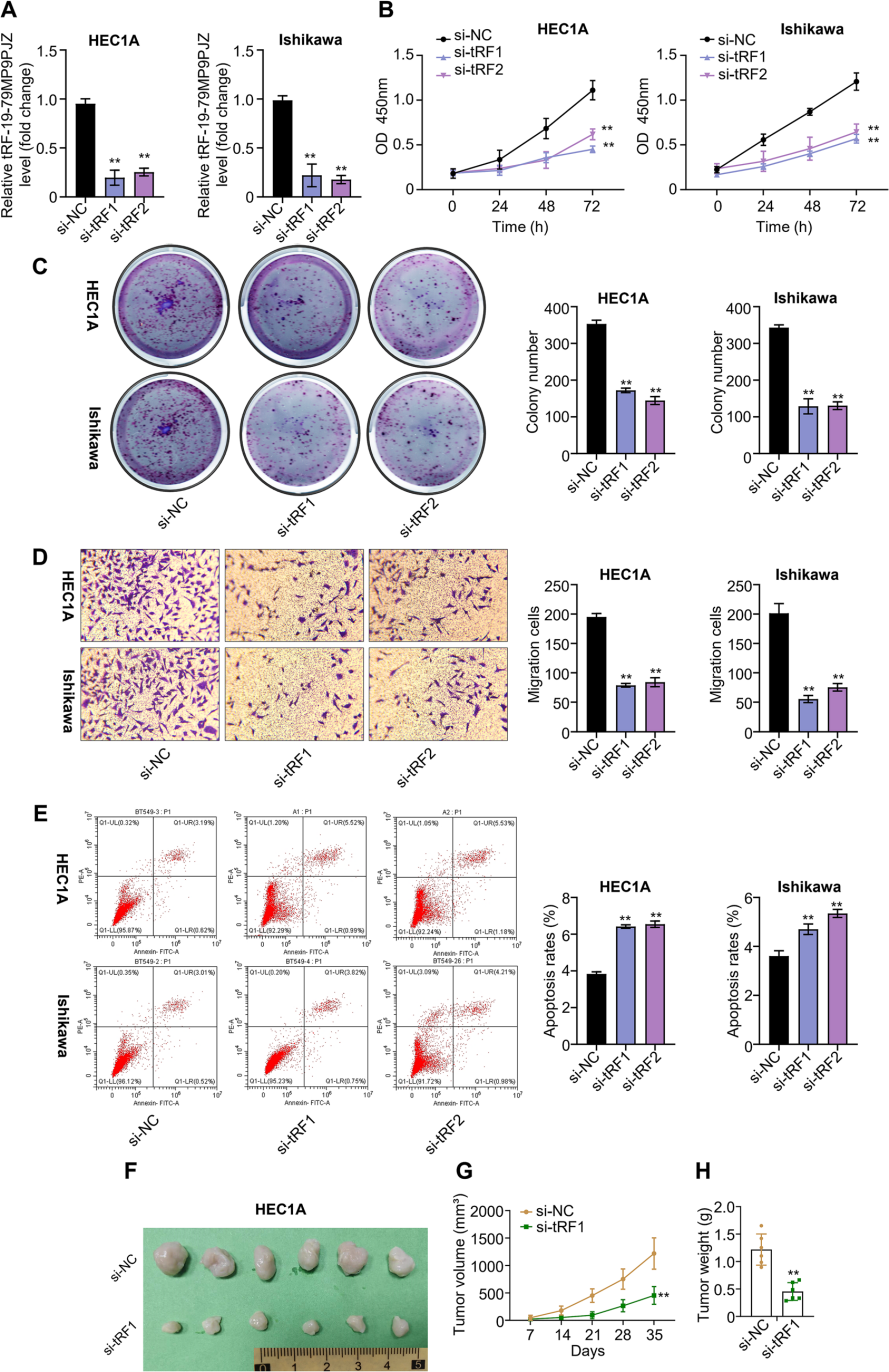
Silencing of tRF-19-79MP9PJZ inhibits proliferation and migration ability and promotes apoptosis in UCEC cell lines. **A** Two siRNAs were designed to knock down tRF-19-79MP9PJZ in HEC1A and Ishikawa cell lines, with knockdown efficiency evaluated by qRT-PCR following transfection. Subsequent analyses included assessment of cell viability via CCK-8 assay (**B**), proliferation via colony formation assay (**C**), migration via Transwell assay (**D**), and apoptosis via flow cytometric analysis (**E**). For in vivo experiments, measurements were taken of subcutaneous tumor mass (**F**), tumor volume (**G**), and tumor weight (**H**) in nude mice. Tumor volume was recorded on days 7, 14, 21, 28, and 35, while tumor weight was measured on day 35. **p < 0.01

To investigate the role of tRF-19-79MP9PJZ in UCEC progression, we conducted loss-of-function experiments using two distinct siRNAs (si-tRF1 or si-tRF2) targeting tRF-19-79MP9PJZ in HEC1A and Ishikawa cells. qRT-PCR confirmed the efficacy of tRF-19-79MP9PJZ knockdown, with si-tRF1 or si-tRF2 reducing its expression by approximately 80% compared to the negative control (si-NC) (Fig. 2A). Subsequently, we examined the impact of tRF-19-79MP9PJZ knockdown on the viability, proliferation, migration, and apoptosis of UCEC cells. The CCK-8 assay, colony formation assay, and Transwell assay demonstrated that tRF-19-79MP9PJZ knockdown significantly suppressed cell viability (Fig. 2B), clonogenic capacity (Fig. 2C), and migratory capacity (Fig. 2D). Conversely, flow cytometry analysis was employed to assess the effect of tRF-19-79MP9PJZ knockdown on cell apoptosis, and the results demonstrated that knockdown of tRF-19-79MP9PJZ significantly induced apoptosis in HEC1A and Ishikawa cells (Fig. 2E). Furthermore, HEC1A cells with or without tRF-19-79MP9PJZ knockdown were injected into nude mice to establish subcutaneous xenograft models. In the murine model, tRF-19-79MP9PJZ knockdown resulted in reduced tumor size (Fig. 2F), tumor volume (Fig. 2G), and tumor weight (Fig. 2H), indicating that tRF-19-79MP9PJZ is critically required for maintaining UCEC tumor growth in vivo. To further substantiate these findings, we performed complementary gain-of-function experiments by transfecting UCEC cells with tRF-19-79MP9PJZ mimics (tRF-mimics) in HEC1A cells. qRT-PCR confirmed the efficacy of tRF-19-79MP9PJZ overexpression, with tRF-mimics inducing its expression compared to the negative control (tRF-NC) (Fig. S2A). Consistent with its oncogenic role, artificial overexpression of tRF-19-79MP9PJZ significantly enhanced cell viability and clonogenic capacity (Fig. S2B, C). Furthermore, Transwell assays demonstrated a marked increase in migratory potential in tRF-mimics-transfected cells compared to the negative control (Fig. S2D). Flow cytometric analysis concurrently revealed that overexpression of tRF-19-79MP9PJZ protected cells from apoptosis (Fig. S2E). These gain-of-function results mirror our knockdown data, compellingly demonstrating that tRF-19-79MP9PJZ is sufficient to drive the malignant phenotype of UCEC cells. Collectively, these findings underscore the crucial role of tRF-19-79MP9PJZ in UCEC pathogenesis.

### DSC3 is down-regulated and regulated by tRF-19-79MP9PJZ in UCEC

**Fig. 3:**
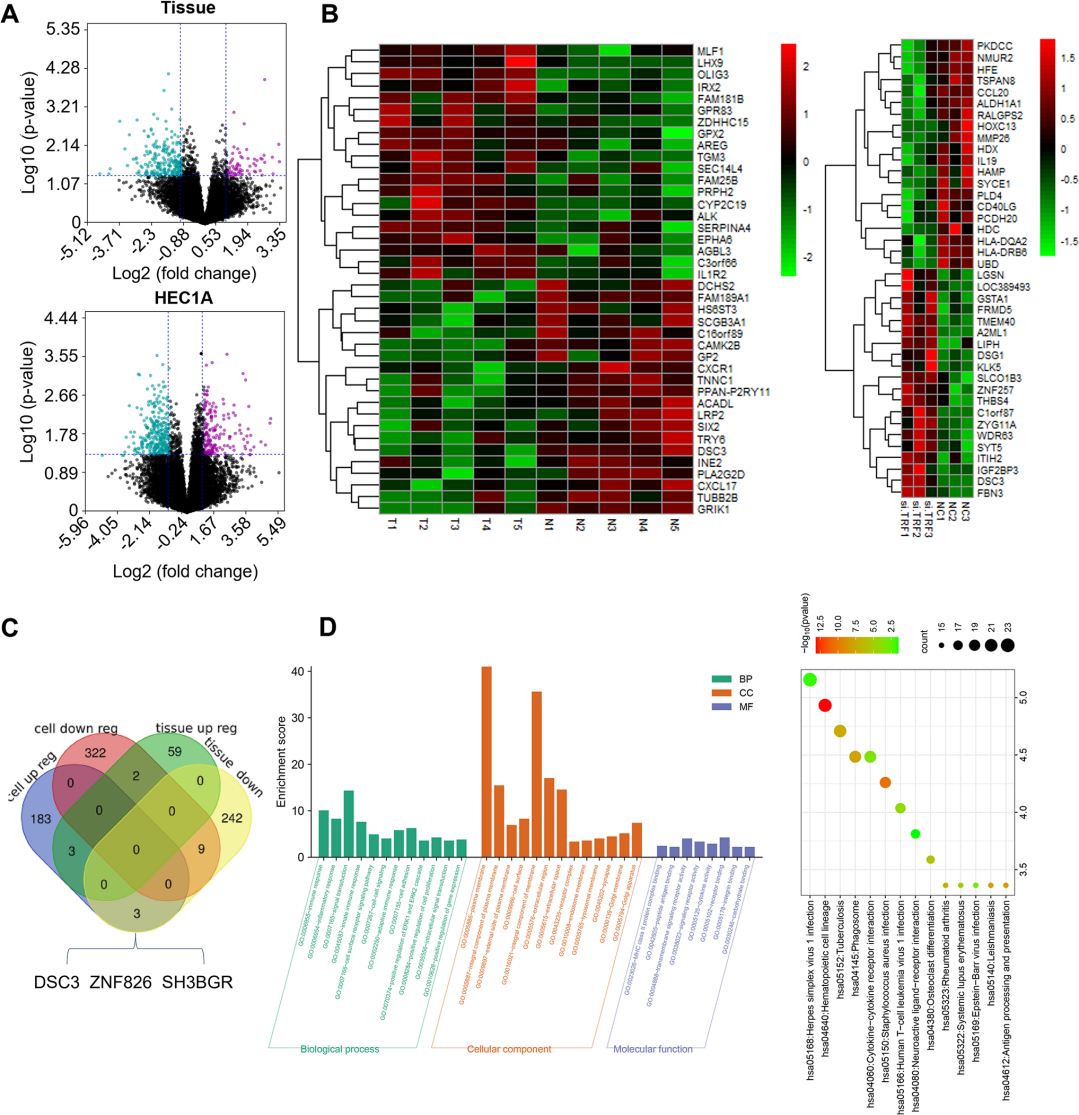
DSC3 is downregulated and regulated by tRF-19-79MP9PJZ in UCEC. Differentially expressed genes between UCEC tumor tissues and adjacent non-tumor tissues, as well as between HEC1A cells with and without tRF-19-79MP9PJZ knockdown, were visualized using volcano plot (**A**) and heatmaps (**B**). **C** Venn diagram analysis illustrating the overlap of upregulated and downregulated genes from the two datasets. **D** Functional enrichment analysis of differentially expressed genes in HEC1A cells with or without tRF-19-79MP9PJZ knockdown

To identify the target genes regulated by tRF-19-79MP9PJZ, we performed transcriptome sequencing on five pairs of UCEC tumor and adjacent non-tumor tissues, as well as HEC1A cells with or without tRF-19-79MP9PJZ knockdown. Figure 3A displays the differentially expressed genes between UCEC tumor and adjacent tissues, and between tRF-19-79MP9PJZ knockdown HEC1A cells and wild-type HEC1A cells. Genes with significant downregulation or upregulation (|logFC|> 1,* p* < 0.05) are visualized in a heatmap (Fig. 3B). Additionally, Venn diagram analysis was performed (Fig. 3C) to compare the sets of downregulated and upregulated genes. Our results revealed that DSC3, ZNF826, and SH3BGR were downregulated in UCEC tumor tissues while being upregulated in HEC1A cells with tRF-19-79MP9PJZ knockdown. To further elucidate the functional implications of these differentially expressed genes, we analyzed the biological processes and signaling pathways associated with the gene expression alterations observed in HEC1A cells (Fig. 3D). This analysis yielded valuable insights into the potential downstream pathways regulated by tRF-19-79MP9PJZ. Collectively, our findings underscore the role of tRF-19-79MP9PJZ in regulating gene expression, with a particular emphasis on its involvement in the downregulation of DSC3, ZNF826, and SH3BGR in UCEC.

### tRF-19-79MP9PJZ reduces DSC3 expression by directly targeting its 3’-UTR in UCEC

**Fig. 4:**
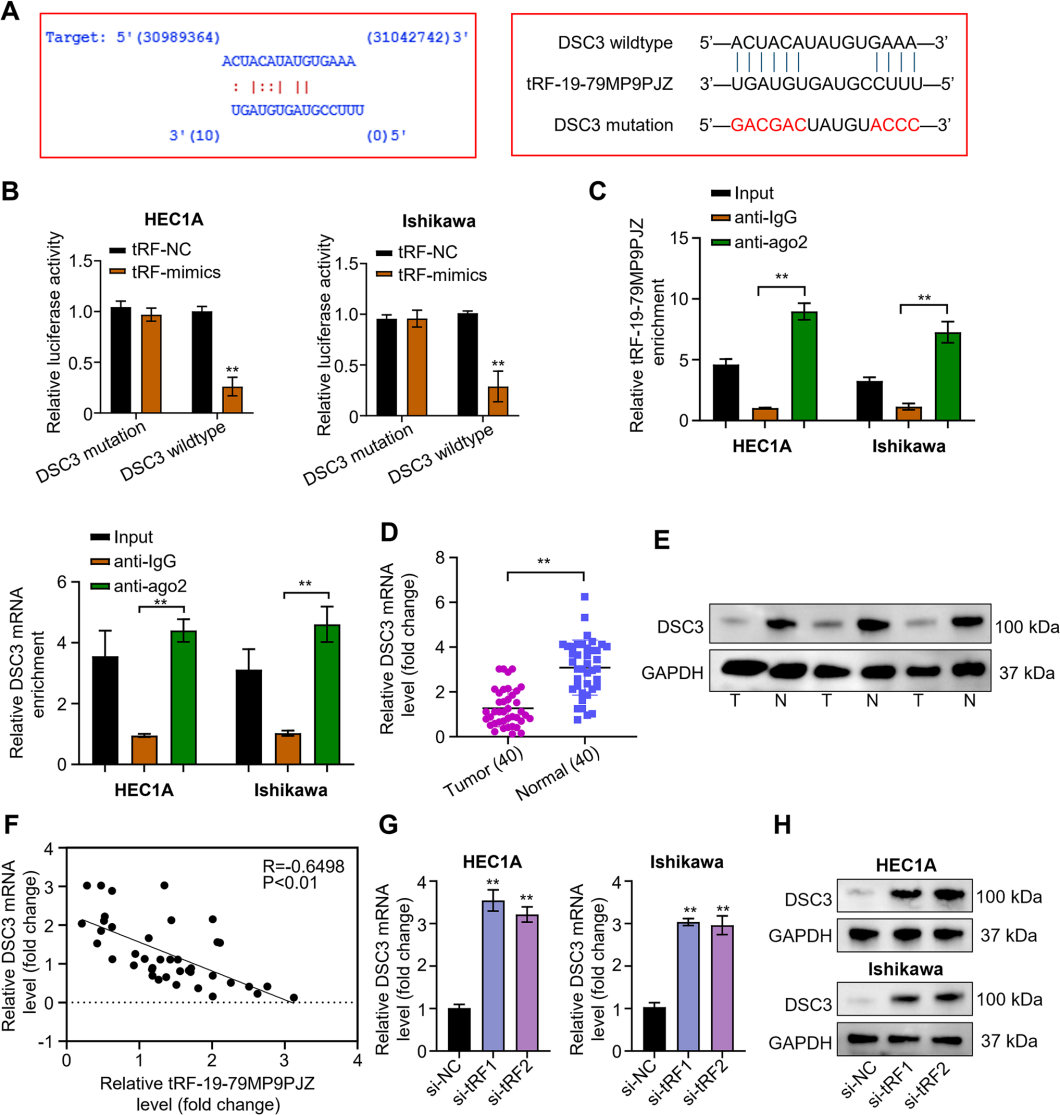
tRF-19-79MP9PJZ regulates DSC3 expression by directly targeting the 3’-UTR of DSC3. **A** Bioinformatic prediction via the tsRFun database indicating the binding of tRF-19-79MP9PJZ to the 3’UTR of DSC3, along with the mutation sites utilized in binding validation assays. **B** Dual-luciferase reporter assay performed in HEC1A and Ishikawa cells following co-transfection of wildtype or mutant DSC3 3’-UTR reporter constructs with tRF-19-79MP9PJZ mimics (tRF-mimics) or negative control (tRF-NC). **C** RIP-qPCR experiment demonstrating efficient enrichment of tRF-19-79MP9PJZ and DSC3 mRNA on AGO2 protein. **D** qRT-PCR analysis of DSC3 expression in 40 collected UCEC patient samples. **E** Western blot analysis of DSC3 protein expression in UCEC patient samples. **F** Correlation analysis revealing a negative correlation between DSC3 mRNA level and tRF-19-79MP9PJZ expression in UCEC. **G**, **H** qRT-PCR and western blot analyses to detect DSC3 expression in HEC1A and Ishikawa cells following tRF-19-79MP9PJZ knockdown. **p < 0.01

To investigate the molecular mechanism underlying the role of tRF-19-79MP9PJZ in UCEC, we first predicted its target genes using tsRFun. As shown in Fig. 4A, tRF-19-79MP9PJZ harbors a sequence complementary to the 3’UTR of DSC3. Notably, ZNF826 and SH3BGR were not predicted to directly interact with tRF-19-79MP9PJZ. Subsequently, dual-luciferase reporter assay was performed in HEC1A and Ishikawa cells following co-transfection with reporter plasmids containing either the wild-type or mutated Desmocollin3 3’-UTR along with tRF-19-79MP9PJZ mimics (tRF-mimics) or negative control (tRF-NC). Overexpression of tRF-19-79MP9PJZ significantly reduced luciferase activity in the wild-type reporter construct, whereas no significant change was observed in the mutant construct (Fig. 4B). These results confirmed the direct binding of tRF-19-79MP9PJZ to the 3’-UTR of DSC3. Additionally, RIP-qPCR experiment performed to assess the direct interaction between tRF-19-79MP9PJZ and DSC3, ZNF826, or SH3BGR. The data showed that both tRF-19-79MP9PJZ and DSC3 were enriched in the Ago2 immunoprecipitates (Fig. 4C), whereas ZNF826 and SH3BGR were not (Figure S1A). These lines of evidence demonstrate that tRF-19-79MP9PJZ is capable of directly binding to DSC3. While regulatory relationships between tRF-19-79MP9PJZ and ZNF826/SH3BGR are observed, such interactions may be mediated through intermediate pathways. Therefore, our subsequent investigations focused primarily on the direct regulatory mechanism between tRF-19-79MP9PJZ and DSC3.

Furthermore, qRT-PCR (Fig. 4D) and western blot analyses (Fig. 4E) were performed to determine the expression levels of DSC3 in 40 collected UCEC tissues. These analyses revealed that DSC3 expression was downregulated in UCEC tumor tissues. Finally, correlation analysis demonstrated a negative correlation between the mRNA levels of DSC3 and tRF-19-79MP9PJZ in UCEC patient tissues (Fig. 4F). To further clarify the specific interaction between tRF-19-79MP9PJZ and DSC3, we assessed DSC3 expression in HEC1A and Ishikawa cells with tRF-19-79MP9PJZ knockdown or overexpression using qRT-PCR and western blot analysis. The results showed that after the expression of tRF-19-79MP9PJZ was knocked down, the expression of DSC3 was upregulated at both mRNA and protein levels (Fig. 4G, H). On the contrary, when tRF-19-79MP9PJZ was overexpressed, the mRNA and protein levels of DSC3 were significantly downregulated (Fig. S1B–C). Collectively, these findings provide robust evidence that tRF-19-79MP9PJZ regulates DSC3 expression by directly targeting the 3’-UTR of DSC3.

### tRF-19-79MP9PJZ promotes UCEC tumor progression by targeting DSC3

**Fig. 5:**
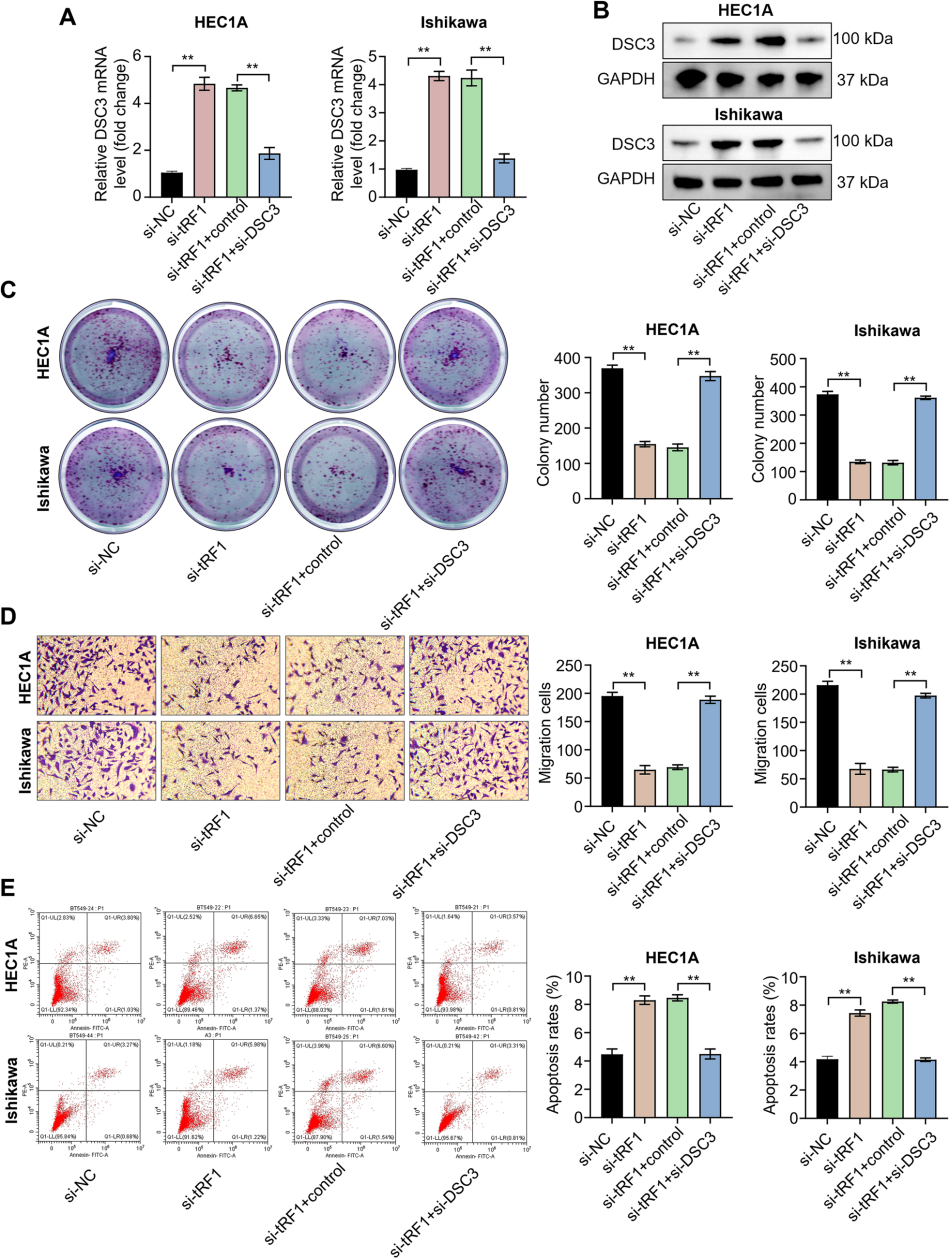
tRF-19-79MP9PJZ promotes UCEC tumor progression by targeting DSC3. **A**, **B** qRT-PCR and western blot analyses were performed to detect DSC3 expression in HEC1A and Ishikawa cells following tRF-19-79MP9PJZ knockdown, with or without DSC3 silencing (co-transfection with si-tRF and si-DSC3). **C** Clone formation assay was conducted to evaluate the clonogenic capacity of HEC1A and Ishikawa cells co-transfected with si-tRF and si-DSC3. **D** Transwell assay was employed to assess the migratory capacity of HEC1A and Ishikawa cells co-transfected with si-tRF and si-DSC3. **E** Flow cytometric analysis was utilized to determine the changes in the apoptotic percentage of HEC1A and Ishikawa cells co-transfected with si-tRF and si-DSC3. **p < 0.01

To further investigate the molecular mechanism of tRF-19-79MP9PJZ involved in UCEC progression, rescue experiments were performed by silencing DSC3 in tRF-19-79MP9PJZ knockdown cells (si-tRF). qRT-PCR and Western blot analyses revealed that knockdown of tRF-19-79MP9PJZ significantly upregulated DSC3 expression, whereas co-transfection with si-DSC3 abrogated this effect (Fig. 5A and B). Subsequently, colony formation assay was performed to assess the clonogenic capacity of UCEC cells. Compared with control cells, tRF-19-79MP9PJZ knockdown cells exhibited a reduced potential for colony formation, and this effect was reversed by co-transfection with si-DSC3. These results indicate that tRF-19-79MP9PJZ regulates tumor proliferation via DSC3 (Fig. 5C). Subsequently, Transwell assay was performed to assess the migratory capacity of UCEC cells. Consistent with the results of the colony formation assay, tRF-19-79MP9PJZ knockdown cells exhibited a diminished migratory potential, and this phenotype was rescued by co-transfection with si-DSC3 (Fig. 5D). Finally, flow cytometry analysis revealed that silencing of tRF-19-79MP9PJZ promotes apoptosis in UCEC cells, whereas co-transfection with si-DSC3 reduced the apoptotic rate (Fig. 5E). Collectively, these findings provide compelling evidence that tRF-19-79MP9PJZ facilitates UCEC tumor progression through the downregulation of DSC3.

## Discussion

UCEC ranks among the most common malignancies in women, with its incidence having increased by 132% over the past three decades. It is frequently diagnosed at advanced stages, and the median age of affected patients is 61 years, with a 5-year survival rate of less than 20% [1–6]. Consequently, there is an urgent need for novel diagnostic and therapeutic strategies for the management of UCEC.

tRFs are increasingly recognized as potentially important regulators in oncogenesis 13–16. Our study provides valuable insights into the involvement of tRFs in UCEC progression, highlighting tRF-19-79MP9PJZ as a critical driver of UCEC malignancy. Our loss-of-function and in vitro gain-of-function experiments suggest that tRF-19-79MP9PJZ is critically involved in the proliferation, migration, and metastasis of UCEC cells while suppressing apoptosis, which underscores its pivotal role in UCEC pathogenesis. Therefore, therapeutic strategies targeting tRF-19-79MP9PJZ may offer a more precise approach, with the potential to enhance treatment efficacy and reduce adverse effects.

We identified DSC3, a member of the cadherin superfamily [24], as a direct downstream target of tRF-19-79MP9PJZ. DSC3 is critical for cell adhesion and the maintenance of desmosomal integrity. In UCEC and other malignancies, DSC3 is frequently downregulated, which is consistent with its role as a tumor suppressor. The interaction between tRF-19-79MP9PJZ and the 3’-UTR of DSC3 mRNA results in the downregulation of DSC3, thereby perturbing cell adhesion dynamics. This perturbation enhances cancer cell proliferation and metastasis, providing a mechanistic basis for the observed effects of tRF-19-79MP9PJZ on UCEC progression.

By expanding the functional landscape of tRFs in cancer biology, our research provides novel insights into the roles of tRFs beyond their canonical functions in translation regulation. Furthermore, considering the potential role of tRF-19-79MP9PJZ in UCEC progression, it may hold certain clinical implications. First, tRF-19-79MP9PJZ exhibits considerable potential as a diagnostic and prognostic biomarker for the early detection and risk stratification of UCEC. Secondly, the development of tRF-19-79MP9PJZ inhibitors could pave the way for innovative therapeutic strategies. Nevertheless, our study is not without limitations that necessitate further investigation. Although DSC3 has been validated as a downstream target, our comprehension of the tRF-19-79MP9PJZ regulatory network remains incomplete. Additional studies are imperative to unravel other potential targets and pathways associated with tRF-19-79MP9PJZ. Moreover, while our loss-of-function in vivo models demonstrate the necessity of tRF-19-79MP9PJZ for tumor growth, the lack of in vivo gain-of-function data limits our ability to fully assert its tumor-promoting capabilities. Future expanded in vivo investigations, including overexpression models, are required to validate these findings and evaluate the therapeutic potential of targeting the tRF-19-79MP9PJZ/DSC3 axis. Furthermore, given that our current study primarily focuses on correlative analyses, future research is warranted to establish a definitive causal relationship between tRF-19-79MP9PJZ and UCEC progression.

In conclusion, our study demonstrates that tRF-19-79MP9PJZ plays a critical role in supporting UCEC tumorigenesis through the downregulation of DSC3 expression. This work not only enhances our understanding of UCEC biology but also identifies novel potential targets for targeted therapeutic strategies.

## Supplementary Information

Below is the link to the electronic supplementary material.

Supplementary file1 (DOCX 784 KB)

Supplementary file2 (PDF 385 KB)

## Data Availability

The datasets used and/or analyzed during the current study are available from the corresponding author upon reasonable request.
