# Supplementary material for: A novel tRNA-derived fragment, tRF-19-79MP9PJZ, promotes uterine corpus endometrial carcinoma progression by targeting DSC3
Source: Hum Cell. 2026 Aug 6;39(8):122. doi: 10.1007/s13577-026-01432-x (PMC13447338; doi:10.1007/s13577-026-01432-x)
Supplement: Supplementary file 1 [file 13577_2026_1432_MOESM2_ESM.pdf]

DSC3
100 kDa

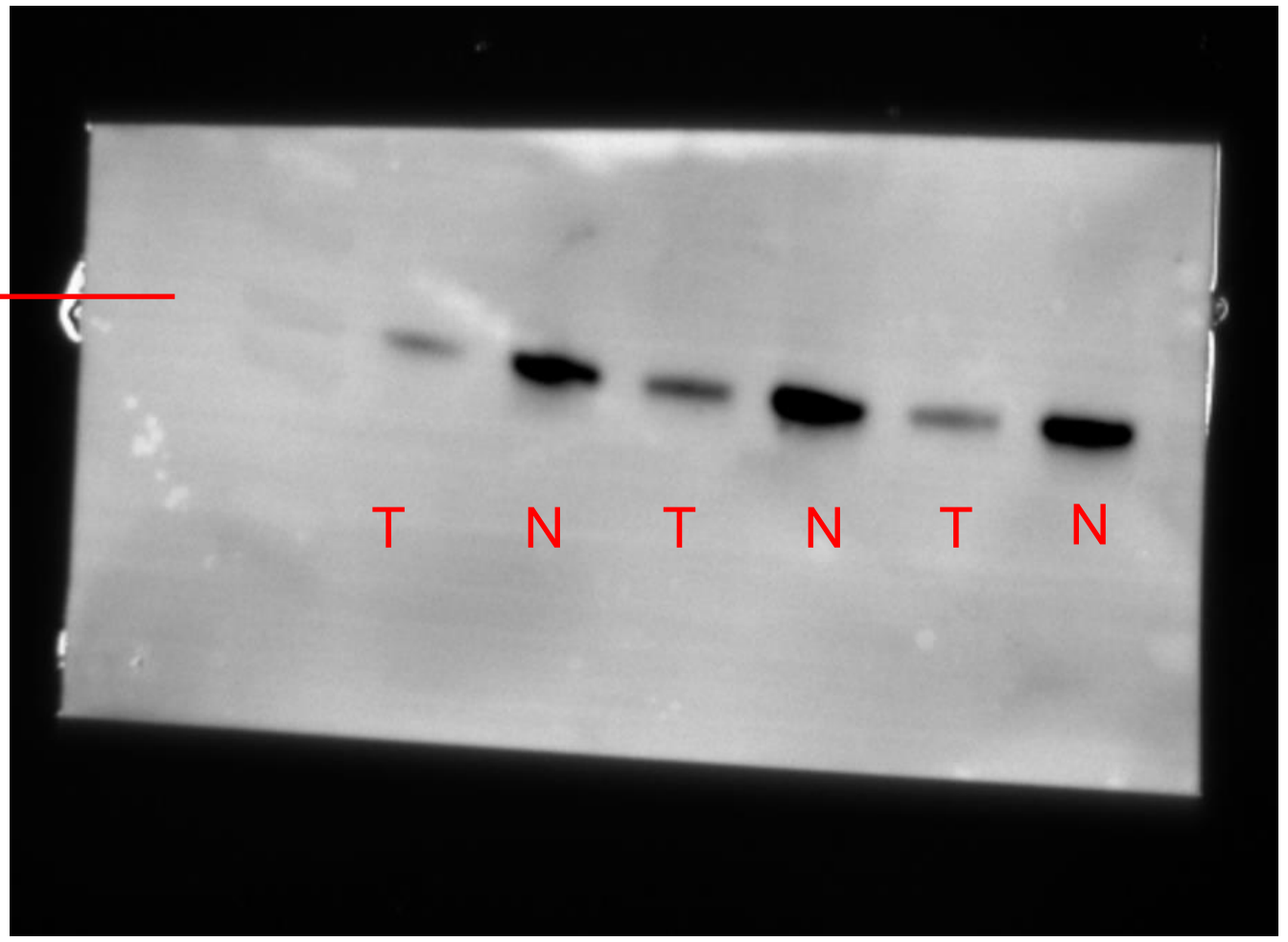

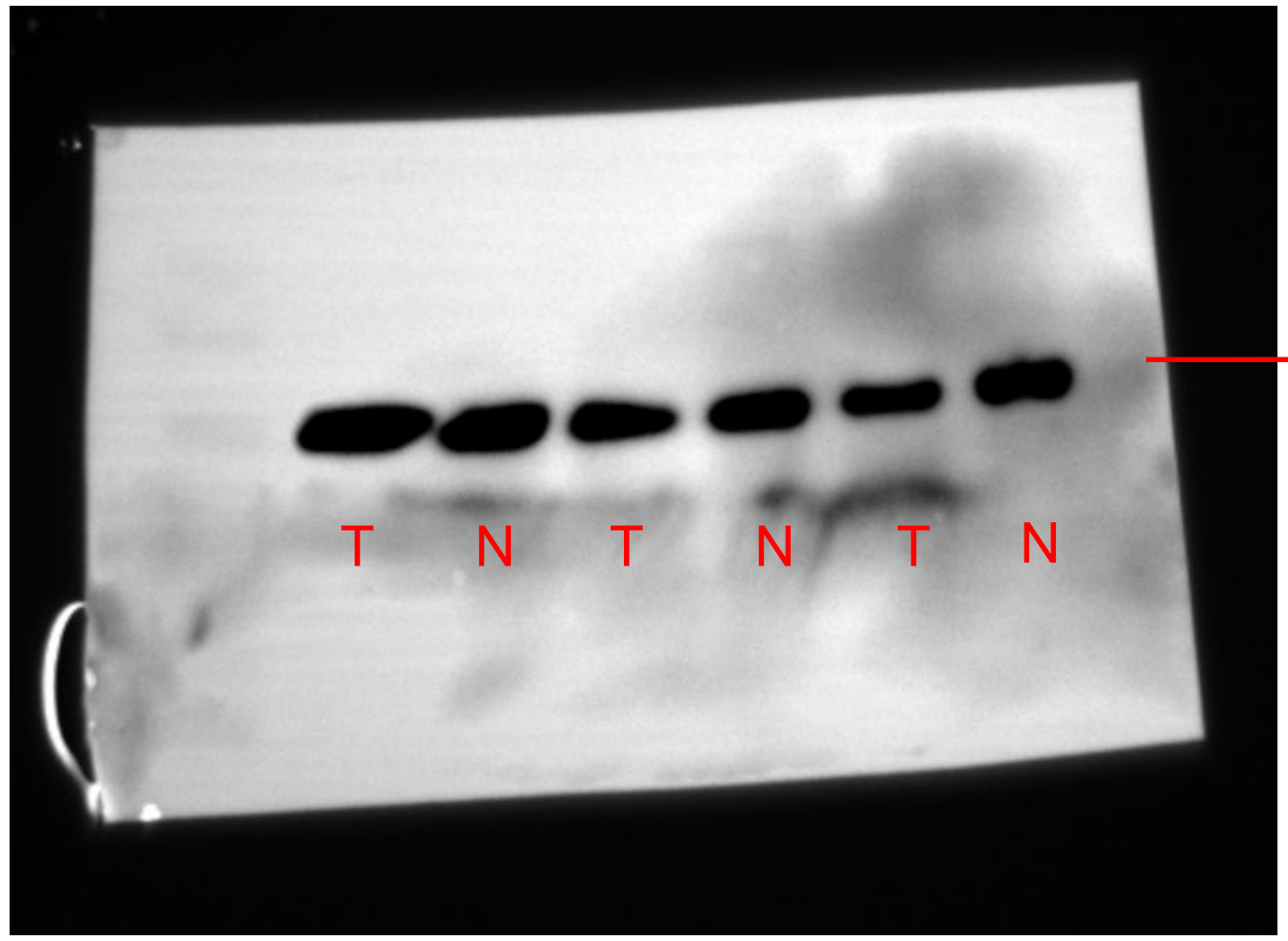

GAPDH
37 kDa

Raw data of blots for Figure 4E

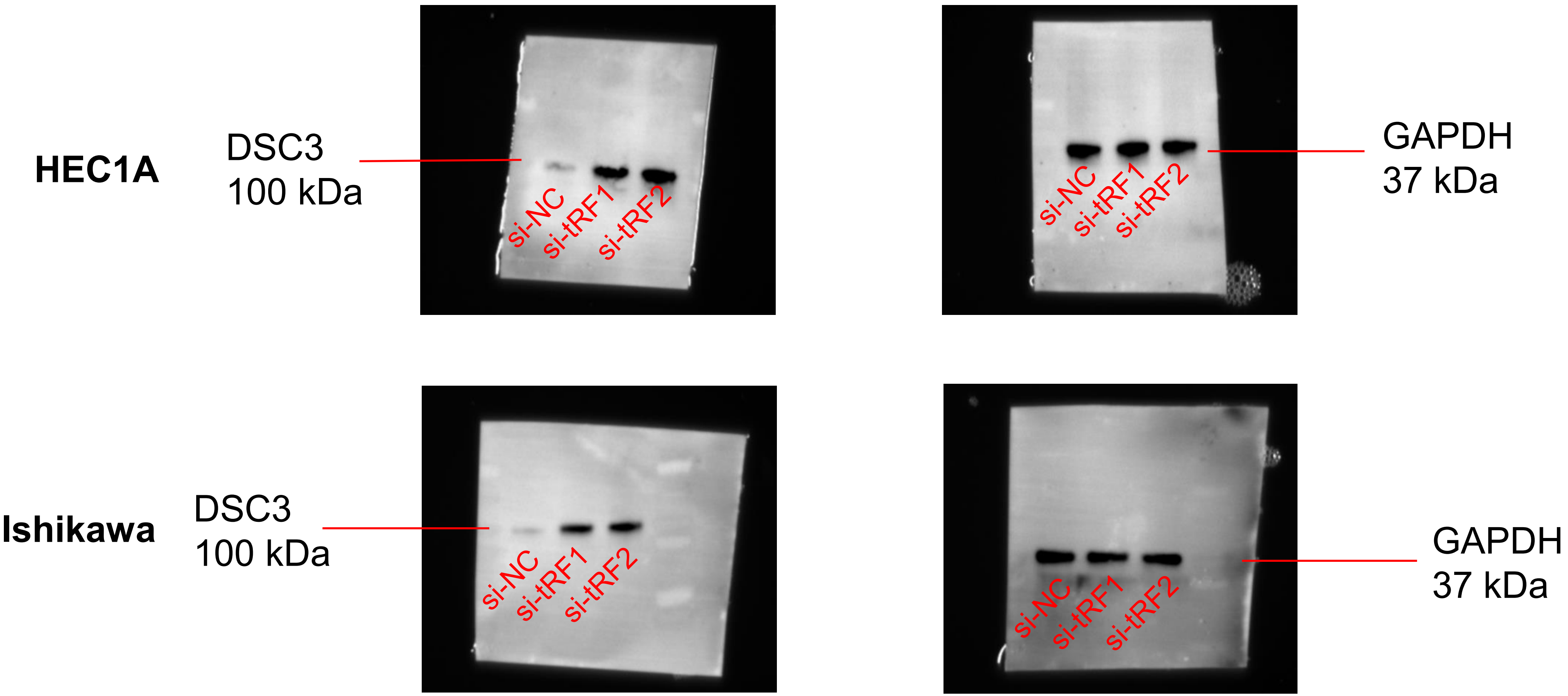

Raw data of blots for Figure 4H

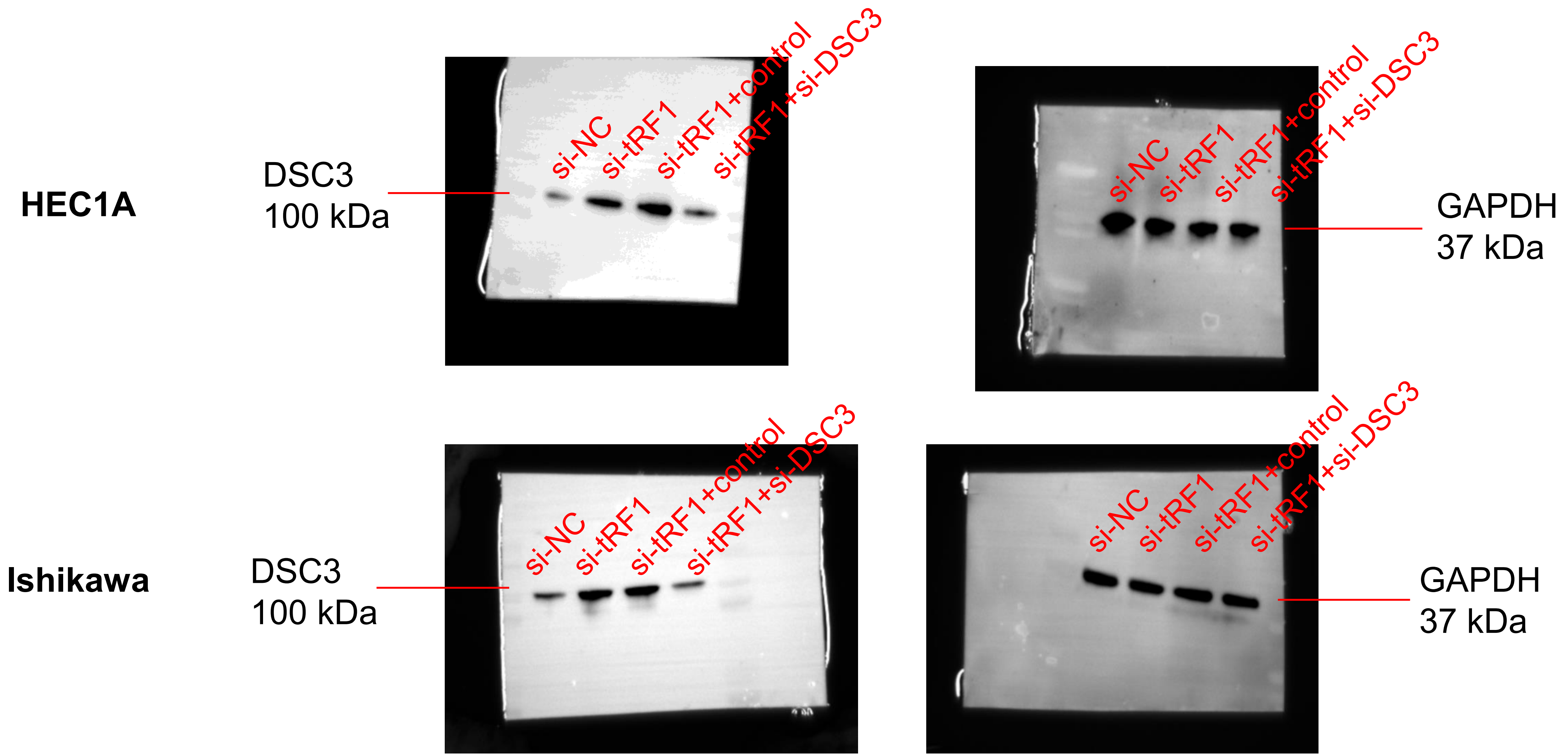

Raw data of blots for Figure 5B

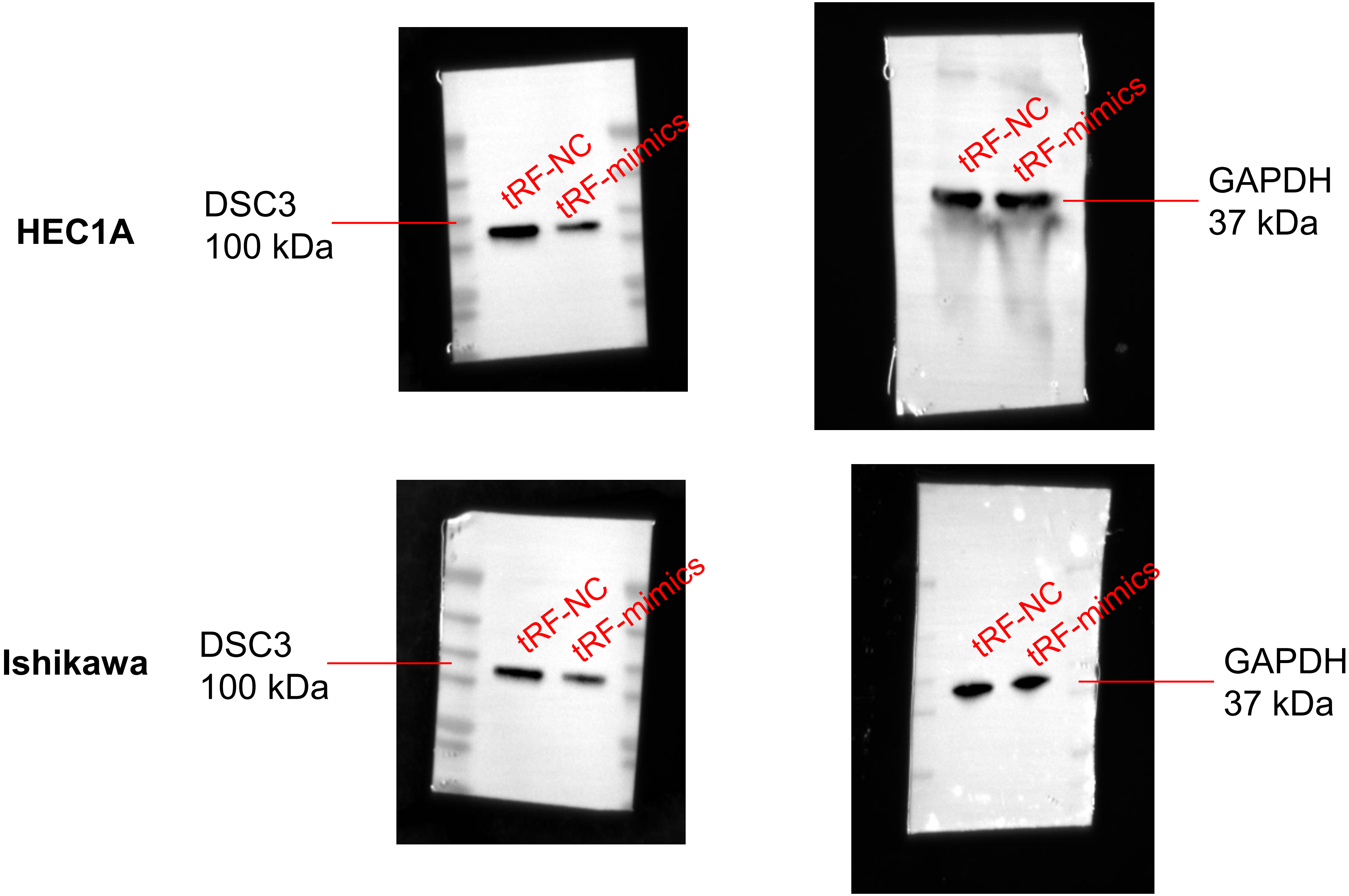

Raw data of blots for Figure S1C
