# Supplementary material for: A novel tRNA-derived fragment, tRF-19-79MP9PJZ, promotes uterine corpus endometrial carcinoma progression by targeting DSC3
Source: Hum Cell. 2026 Aug 6;39(8):122. doi: 10.1007/s13577-026-01432-x (PMC13447338; doi:10.1007/s13577-026-01432-x)
Supplement: Supplementary file 2 [file 13577_2026_1432_MOESM1_ESM.docx]

**Figure S1.** (A) RIP-qPCR experiment detects effective enrichment of ZNF826 and SH3BGR mRNA on Ago2 protein. (B-C) qRT-PCR and western blot were used to detect DSC3 expression in HEC1A and Ishikawa cells after overexpressing tRF-19-79MP9PJZ. ***p* < 0.01.

**Figure S2.** (A) tRF-NC was designed to overexpress tRF-19-79MP9PJZ in HEC1A cells, with overexpression efficiency evaluated by qRT-PCR following transfection. Subsequent analyses included assessment of cell viability via CCK-8 assay (B), proliferation via colony formation assay (C), migration via Transwell assay (D), and apoptosis via flow cytometric analysis (E). ***p* < 0.01.

**Table S1. Prognostic evaluation of the top 12 up-regulated tRFs in the TCGA UCEC cohort.**

| **tRF ID** | **Overall survival**  **(p-value)** | **Disease-free survival**  **(p-value)** | **Relapse-free survival**  **(p-value)** |
| --- | --- | --- | --- |
| tRF-19-79MP9PJZ | 0.027 | 0.039 | 0.019 |
| tRF-16-6978WP0 | 0.048 | 0.057 | 0.043 |
| tRF-16-73FEWSD | 0.125 | 0.187 | 0.106 |
| tRF-16-90Q4R90 | 0.085 | 0.106 | 0.074 |
| tRF-16-RPM830D | 0.14 | 0.225 | 0.262 |
| tRF-16-K58Y01B | 0.202 | 0.185 | 0.241 |
| tRF-16-K5YY01B | 0.362 | 0.321 | 0.280 |
| tRF-16-2WMK81B | 0.065 | 0.095 | 0.089 |
| tRF-17-8647O52 | 0.176 | 0.157 | 0.184 |
| tRF-17-ME6KM8N | 0.031 | 0.058 | 0.062 |
| tRF-16-S3M830E | 0.127 | 0.184 | 0.210 |
| tRF-16-87R8WPE | 0.699 | 0.584 | 0.618 |
